# A Web-Based Computer-Tailored Alcohol Prevention Program for Adolescents: Cost-Effectiveness and Intersectoral Costs and Benefits

**DOI:** 10.2196/jmir.5223

**Published:** 2016-04-21

**Authors:** Ruben MWA Drost, Aggie TG Paulus, Astrid F Jander, Liesbeth Mercken, Hein de Vries, Dirk Ruwaard, Silvia MAA Evers

**Affiliations:** ^1^ Department of Health Services Research School for Public Health and Primary Care (CAPHRI) Faculty of Health, Medicine and Life Sciences, Maastricht University Maastricht Netherlands; ^2^ Department of Health Promotion School for Public Health and Primary Care (CAPHRI) Faculty of Health, Medicine and Life Sciences, Maastricht University Maastricht Netherlands; ^3^ Trimbos, Netherlands Institute of Mental Health and Addiction Utrecht Netherlands

**Keywords:** adolescents, alcohol use, cluster randomized controlled trial, game, computer tailoring, education, criminal justice, costs and cost analysis, economic evaluation, intersectoral costs and benefits

## Abstract

**Background:**

Preventing excessive alcohol use among adolescents is important not only to foster individual and public health, but also to reduce alcohol-related costs inside and outside the health care sector. Computer tailoring can be both effective and cost-effective for working with many lifestyle behaviors, yet the available information on the cost-effectiveness of computer tailoring for reducing alcohol use by adolescents is limited as is information on the costs and benefits pertaining to sectors outside the health care sector, also known as intersectoral costs and benefits (ICBs).

**Objective:**

The aim was to assess the cost-effectiveness of a Web-based computer-tailored intervention for reducing alcohol use and binge drinking by adolescents from a health care perspective (excluding ICBs) and from a societal perspective (including ICBs).

**Methods:**

Data used were from the Alcoholic Alert study, a cluster randomized controlled trial with randomization at the level of schools into two conditions. Participants either played a game with tailored feedback on alcohol awareness after the baseline assessment (intervention condition) or received care as usual (CAU), meaning that they had the opportunity to play the game subsequent to the final measurement (waiting list control condition). Data were recorded at baseline (T0=January/February 2014) and after 4 months (T1=May/June 2014) and were used to calculate incremental cost-effectiveness ratios (ICERs), both from a health care perspective and a societal perspective. Stochastic uncertainty in the data was dealt with by using nonparametric bootstraps (5000 simulated replications). Additional sensitivity analyses were conducted based on excluding cost outliers. Subgroup cost-effectiveness analyses were conducted based on several background variables, including gender, age, educational level, religion, and ethnicity.

**Results:**

From both the health care perspective and the societal perspective for both outcome measures, the intervention was more costly and more effective in comparison with CAU. ICERs differed for both perspectives, namely €40 and €79 from the health care perspective to €62 and €144 for the societal perspective per incremental reduction of one glass of alcohol per week and one binge drinking occasion per 30 days, respectively. Subgroup analyses showed, from both perspectives and for both outcome measures, that the intervention was cost-effective for older adolescents (aged 17-19 years) and those at a lower educational level and, from a health care perspective, the male and nonreligious adolescent subgroups.

**Conclusions:**

Computer-tailored feedback could be a cost-effective way to target alcohol use and binge drinking among adolescents. Including ICBs in the economic evaluation had an impact on the cost-effectiveness results of the analysis. It could be worthwhile to aim the intervention specifically at specific subgroups.

**Trial Registration:**

Nederlands Trial Register: NTR4048; http://www.trialregister.nl/trialreg/admin/rctview.asp?TC=4048 (Archived by Webcite at http://www.webcitation.org/6c7omN8wG)

## Introduction

Excessive alcohol use and alcohol use disorders are major causes of death and disability worldwide [ 1- 4]. In 2012, approximately 3.3 million deaths, or 5.9% of all global deaths, were attributable to alcohol use [ 4]. In addition, alcohol use led to an estimated total of 139 million disability-adjusted life years, representing 5.1% of the global burden of disease and injury in that year [ 4]. For all age groups, outliers on the proportion of alcohol-related deaths can be seen in the World Health Organization European Region, varying from 10% for the population aged 80 years and older to 25% for the 20 to 39 age group. It is particularly striking that in this region 10% of adolescent deaths (those aged 15 to 19 years) were attributable to alcohol.

Apart from the impact of alcohol use on morbidity and mortality, the harmful use of alcohol may also lead to significant societal costs [ 3- 7]. For example, in the European Union alone, alcohol-attributable costs were estimated at €125 billion in 2003 [ 4]. These encompass health care services, such as hospitalizations, home health care, and ambulatory care, but also costs outside the health care sector, such as costs resulting from productivity losses and costs in the criminal justice system. Examples of the latter include vehicle crashes, increased crime, and arrests. Studies have shown that youthful drinkers are at greater risk of being victimized and perpetrating youth violence, low educational attainment, and low college expectations [ 8, 9], putting a financial burden on the criminal justice system and educational sector.

Preventing excessive alcohol use in the whole population and in the young population in particular is thus important, not only to improve the health of individuals and of the whole population, but also to reduce alcohol-related costs inside and outside the health care sector. Computer tailoring could be a means to achieve these goals. Computer tailoring is a behavioral intervention that can be effective in changing health behaviors in general, including the use of alcohol [ 10- 15]. Within a computer-tailored intervention, the content is adapted to individual characteristics of respondents [ 16]. Often a questionnaire is used as a screening instrument, assessing behavior, relevant sociodemographics, and motivational factors [ 17, 18]. Respondent answers are collected into a data file and automatically matched with tailored feedback messages [ 16, 19]. An advantage of tailored information is that it is perceived as more relevant than nontailored information [ 20, 21]. Moreover, through the Internet, these programs are accessible by a growing percentage of the world population—42.3% had access to Internet in 2014 in comparison to 5.9% in 2000 [ 22]—and can be accessed wherever and whenever it suits the respondents. For Europe and its younger population (aged 16-24 years), the current Internet penetration is even higher: 70.5% [ 22] and 91.0% [ 23], respectively. Taking advantage of this high accessibility by providing Internet-based behavior interventions might significantly limit the need for and burden on health professionals and could, in turn, lead to less administrative costs [ 19]. Subsequently, the resulting savings of time and resources contribute to a more efficient health care system.

Although the effectiveness of multiple Internet-based and computer-tailored interventions have been studied [ 24, 25], limited information exists on their cost-effectiveness [ 26]. To date, studies which have included cost-effectiveness analyses (CEAs) focused on adults aged 18 to 65 years [ 21, 27] and 18 to 69 years [ 3], whereas the cost-effectiveness of such interventions for adolescents younger than 18 years has not yet been studied. Available economic analyses of interventions aimed at reducing alcohol use among adolescents are those of interventions which are not Internet-based and not computer-tailored [ 28, 29]. Furthermore, as acknowledged by Smit et al [ 3], these studies show a limitation in terms of disregarding many of the costs and benefits in sectors outside the health care sector, also known as intersectoral costs and benefits (ICBs) [ 30]. Excluding ICBs, such as costs and benefits in the educational and criminal justice sector, may significantly affect the results of the CEAs of interventions [ 30]. Moreover, including and reporting ICBs within economic evaluations could support decision making regarding the large-scale implementation of such programs. Therefore, the aim of this study is to answer the question of whether a Web-based computer-tailored intervention for adolescents for reducing the use of alcohol is cost-effective from both a health care and societal perspective, and to assess the impact of including ICBs on the outcomes of the analysis.

## Methods

### Design

Data used was from the Alcoholic Alert study, which was designed as a cluster randomized controlled trial (RCT) with randomization at the level of schools into two conditions [ 31]. Participants either played a game on alcohol awareness after the baseline assessment (intervention condition) or received care as usual (CAU), meaning they had the opportunity to play the game subsequent to the final measurement (waiting list control condition). Providing this opportunity was due to ethical considerations. Data were recorded at baseline (T0=January/February 2014) and after 4 months (T1=May/June 2014). These were used to conduct two sets of comparative CEAs; one was performed from a health care perspective (including health care costs, excluding ICBs) and one from a societal perspective (including both health care costs and ICBs). Because of a rapid change in Dutch government policy on the minimum legal drinking age (ie, 18 years vs 17 years as of January 1, 2014), the abovementioned time frame and start date differed from the original design [ 31]. The Alcoholic Alert study was approved by the Medical Ethics Committee of Atrium Orbis Zuyd (METC number: 12-N-104) and was registered in the Dutch Trial Register (NTR 4048).

### Randomization

Randomization was performed at school level to prevent contamination between participants. Randomization was conducted by drawing lots. After randomization, 21 schools were assigned to the intervention condition and 23 schools were assigned to the control condition.

### Sample

The study population consisted of Dutch adolescents (aged 15-19 years) attending school [ 31]. Participants included students at schools of higher secondary education, lower secondary education, and lower vocational training. To have enough power for the evaluation, a participant target was made based on the following criteria: 10% reduction in binge drinking occasions (ie, for girls, at least four glasses or, for boys, five glasses of alcohol-containing drinks in one occasion) [ 32] during the preceding 30 days between the intervention and control group, with an intraclass correlation of .02, a power of 80%, and a significance level of .05. Furthermore, taking into account the drop in power due to an expected dropout of 50% at follow-up, it was estimated that at least 34 schools should be included at T0 [ 31].

To reach the required number of 34, schools were recruited via several media; schools first received flyers with information about the Alcoholic Alert study, after which they were contacted via telephone and email. If schools enrolled in the study, students of the schools were eligible to participate. However, they could do so only if they provided informed consent by clicking a checkbox, which preceded the Web-based questionnaire at T0 [ 31].

### Intervention

**Figure 1:**
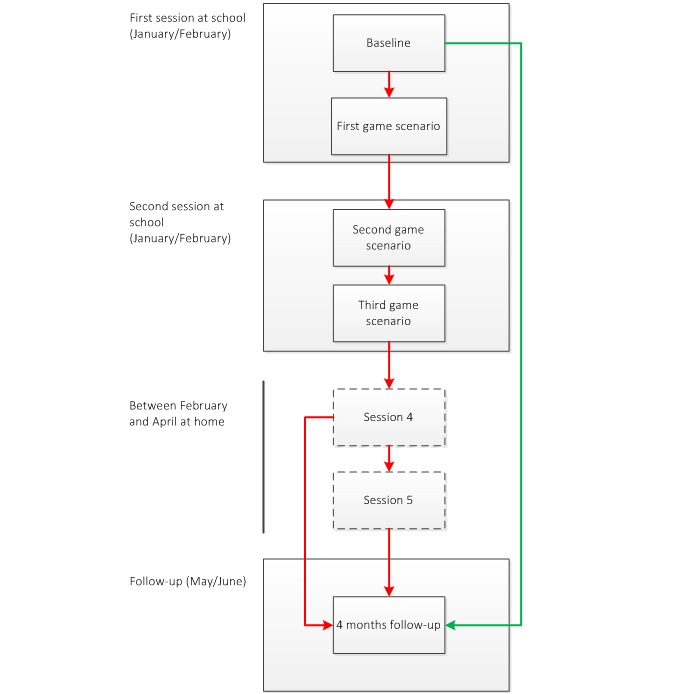
Flowchart of the intervention (based on Jander et al [ 31]). Red line: routing intervention condition; green line: routing control line; dashed boxes: intervention parts that had to be done at home.

Adolescents in the intervention condition participated in a Web-based computer-tailored alcohol reduction program called Alcoholic Alert [ 31]. After completing a Web-based questionnaire on the Alcoholic Alert website at T0, the participants entered a game called “Watskeburt” (Dutch slang for “What Happened?!”). In the game, the participant played a character whose goal it was to find out what happened after a night of heavy drinking. Participants received in-game questions concerning alcohol-related sociocognitive factors, including attitude, social influences, self-efficacy expectations, and action plans toward alcohol drinking. These questions were based on the I-Change model, which is an integrated model explaining motivational and behavioral change [ 33]. Based on their answers, they received computer-tailored feedback on these determinants. They played in three game scenarios within three sessions. A week after playing the third game scenario, the participants were asked to revisit the intervention website to answer several questions. In this fourth session, they were asked about their drinking behavior during the preceding week and then they received computer-tailored feedback on their alcohol use compared to Dutch drinking guidelines. Subsequently, the participants were asked whether they had an event (eg, party, wedding) in the upcoming 30 days then they were challenged to drink less than usual and were asked for the maximum amount they wanted to drink. An email, with a reminder of accepting the challenge, was sent to them a day before the event. Two days after the event, during a fifth session, they were asked to visit the intervention website and fill in their alcohol use. If the participant failed the challenge, they received computer-tailored feedback with tailored advice and had the opportunity to take on a new challenge. If the participant met the challenge, he or she received congratulations and the intervention was over ( Figure 1).

Participants receiving CAU also filled in the Web-based questionnaire at T0 and T1, but they did not have access to the game and did not receive computer-tailored feedback until after the final measurement. Further information on the intervention can be found elsewhere [ 31].

### Measurements and Outcomes

The measurements at T0 and T1 were performed at school after participants received instructions from their teachers [ 31]. The Web-based questionnaires used for the measurements included items related to alcohol drinking behavior, use of services within the health care sector, and ICBs. In addition, several background variables were measured at T0, including gender, age, educational level (higher secondary level, lower secondary level, and lower vocational), religion (Catholic, Protestant, Muslim, other religion, no religion), and ethnicity (Dutch, Antillean, Belgian, German, Surinamese, Moroccan, Turkish, other) [ 31].

In this study, the outcome measures were weekly alcohol use and the number of binge drinking occasions in the preceding 30 days. Weekly alcohol use was assessed by asking participants on which days in the past week they had been drinking and, if they did, how many glasses of alcohol they had on these days. Based on this information, the total amount of glasses of alcohol was calculated [ 17]. From this, as for binge drinking occasions, the weekly alcohol use at T1 was subtracted from the alcohol use at T0. This led to positive scores in case of a reduction in weekly alcohol use or number of binge drinking occasions, and negative scores in case weekly alcohol use or the number of binge drinking occasions increased.

### Resource Use and Costing

The following costs related to the Alcoholic Alert intervention were identified as important and measured: (1) intervention costs, (2) health care costs (ie, costs for services inside the health care sector), (3) intersectoral costs (ie, costs for services outside the health care sector), and (4) costs of substance use (eg, use of hard drugs). Because alcohol use was the outcome measure in this study, the costs of alcohol use were not included in the CEA. Doing so would have led to double counting [ 34].

Costs (in Euros) were measured irrespective of who bore them and were indexed for the reference year 2014 using price indexes from Statistics Netherlands [ 35]. Calculations for specific costs of service utilization and substance use can be obtained from the first author (RD).

#### Intervention Costs

Intervention costs were divided into costs made during the development of the intervention and costs for running the intervention. Costs incurred during the development included game development costs (€20,328) and automatic tailoring software license and development costs (€8367.15). These costs and costs for other personnel involved in the development and application of the intervention for this study, such as costs incurred contacting schools, recruiting participants, and analyzing data are sunk costs [ 36]. Therefore, these are not included in the CEA. This is further justified by the fact that the intervention itself is Web-based and universal for adolescents, meaning the intervention has a wide reach and the development costs per participant drop to a minimum when it is used widely and structurally.

Costs for running the intervention include hosting costs for the website, tailored feedback software, and participants’ time investments. As for the sunk costs, the website hosting costs (€300 per year) per participant drop to a minimum if used widely and are not included in the CEA. Tailored feedback software costs were €7 per participant per week. In case the participant met his or her challenge in the fifth session, he or she would receive tailored feedback over a period of 3 to 6 weeks. The mean intervention duration was 4 weeks, so the tailored feedback costs were €28. The five sessions took up 1.5 hours at school (€8.30 per hour) and 1 hour of free time (€12.50). The total of 2.5 hours was valued at €25 per participant. In sum, the total intervention costs per participant were an estimated €53.

#### Health Care Costs

Health care costs were calculated by multiplying volumes of health services by related cost prices. Health services measured included contacts with the general practitioner, emergency care, hospital stays, ambulance rides, and mental health services. Cost prices were drawn from the Dutch manual for costing in economic evaluations [ 37].

#### Intersectoral Costs

The ICB-related costs were calculated by multiplying volumes of services and time investments outside the health care sector with related cost prices. The ICBs were classified in sectors according to a classification scheme for ICBs by Drost et al [ 30]. The sectors in this scheme included education, labor and social security, household and leisure, and criminal justice system. The services and time investments measured included school absenteeism and contacts with an attendance officer (education), work absenteeism (labor and social security), failing to perform household and other activities, contacts with youth and family center and family care (household and leisure), and contacts with (youth) police services, court proceedings, and child (health) protection services (criminal justice system). Cost prices were drawn from a Dutch manual for intersectoral costs and benefits of (preventive) interventions [ 38]. Cost prices not mentioned in the manual were extracted from the Institute for Medical Technology Assessment (iMTA) questionnaire on intensive youth care [ 39]. For ICBs that required valuation of time, such as failing to perform household activities, some additional information was drawn from the Dutch report “The Netherlands in a Day” (free translation) [ 40].

#### Costs of Substance Use

In addition to alcohol use, use of other substances were measured as well. These included packs of cigarettes, use of soft drugs, and use of hard drugs. Cost prices were found on the website of the Jellinek Clinic, which is a renowned Dutch institution specializing in preventing and treating alcohol and substance abuse [ 41].

### Data Preparation

The basis of the analysis was the dataset used for the Alcoholic Alert effect study [ 24]. However, because cost measures were used for conducting the economic evaluation, some additional data cleaning was required to create a dataset that was suitable for conducting the CEA.

First, because the digital questionnaires contained open-ended questions, participants had the opportunity to fill in unrealistic answers. It was a small subsample that systematically filled in these unrealistic answers, but to improve the validity of the results, these respondents were excluded from analysis. To clear the data of these respondents, limits were set for each variable. Participants breaching these limits by providing unrealistic answers were excluded from analysis. For example, because the 4-month recall period between T0 and T1 amounted to 120 days, any respondent claiming to have stayed more than 120 days in a hospital was excluded. In general, to reduce the chance of wrongfully excluding participants, limits were set high, but within the range of credibility. As for all steps during the process of data preparation and analysis, the limits were discussed in author meetings and were agreed on by all authors. A list of these limits can be obtained in Multimedia Appendix 1.

Second, the dataset was cleared of respondents who at baseline did not answer a single question related to costs. Based on these two steps, the sample at T0, and accordingly at T1, was smaller and different in composition in the CEA than the sample used for the effect study [ 24].

Finally, to assess the school-based part of the variance in this cluster RCT, intracluster correlation coefficients (ICCs) were calculated for both the weekly alcohol use and binge drinking occasions outcome measures. The ICCs were calculated based on the following formula: ICC or ρ = s _b_^2^/(s _b_^2^+ s _w_^2^), where *s*_*b*_^*2*^= the variance between clusters and *s*_*w*_^*2*^= the variance within clusters [ 42]. Input for both outcome effect sizes was generated using SPSS version 20 by running linear regression mixed models. These analyses and corresponding calculations resulted in ρ=.06 for binge drinking occasions and ρ=.01 for weekly alcohol use, which shows that the within-cluster variances for both effect sizes were much greater than the between-cluster variances [ 42]. Based on these results, no re-estimations of effects were required.

### Analysis

#### Descriptive Statistics

Descriptive statistics were used to describe the characteristics of the sample at T0 and at T1. Differences between the intervention and control conditions were assessed in SPSS version 20 using independent samples *t*tests for continuous variables and chi-square tests for discrete variables. The same software was used to conduct stepwise linear regression analyses to assess the dependence of the outcome measures on these variables.

#### Cost-Effectiveness Analysis

The base scenario of this study included CEAs from the two perspectives mentioned earlier. We calculated costs of services utilization in three steps: (1) assessment of the services and time consumed in the 4-month period between T0 and T1, (2) calculation of the associated costs in Euros, and (3) calculation of the incremental cost-effectiveness ratio (ICER) using the formula (C _i_–C _c_)/(E _i_–E _c_). Here *C*represents the average total costs per participant during the 4-month period between T0 and T1 and *E*represents the mean difference in the number of glasses of alcohol or binge drinking occasions at T1 in comparison with the number measured at T0 in the intervention (C _i_and E _i_) and in the control (C _c_and E _c_) condition.

Stochastic uncertainty in the data was dealt with using nonparametric bootstraps. By using the bootstrapping technique, means and confidence intervals were calculated and 5000 ICERs were simulated, which were plotted in cost-effectiveness planes. These planes provided a visual representation of the probability of the intervention being cost-effective in comparison with the control condition by showing the distribution of ICERs across four quadrants: (1) more effective and more costly in the northeast quadrant (NE), (2) more effective and less costly in the southeast quadrant (SE), (3) less effective and less costly in the southwest quadrant (SW), and (4) less effective and more costly in the northwest quadrant (NW) [ 43].

An ICER in the SE and NW is negative, indicating that the intervention is dominant over (SE) or inferior to (NW) the control condition. An ICER in the SW or NE is positive, indicating that from a cost-effectiveness viewpoint the intervention is more favorable than the control condition only when the ICER is lower than the willingness to pay (WTP) per unit effect. Because no threshold (ie, maximum WTP) was available for the weekly alcohol use outcome measure, a cost-effectiveness acceptability curve (CEAC) was created for both perspectives. The CEAC showed the likelihood of the intervention being favorable over CAU for several hypothetical thresholds.

#### Sensitivity and Subgroup Analyses

Apart from the analyses to deal with stochastic uncertainty, several other analyses were conducted. First, to assess the impact of cost outliers, ICERs were calculated based on data in which cost outliers were excluded. Based on the output of descriptive statistics, it was decided to exclude participants when total costs were greater than €5000. Based on this approach, in the analyses that were conducted from the health care perspective, one participant was excluded. In the analyses that were conducted from the societal perspective, four participants were excluded. Second, to assess the effect of the uptake, the costs of cigarette use, and of the use of soft and hard drugs in the analyses conducted from the societal perspective, additional analyses were conducted without these costs.

Finally, given the heterogenic composition of the study sample, several subgroup CEAs were conducted based on the background variables measured at T0. These included analyses based on dichotomized background variables, including gender (male, female), age (15-16, ≥17), educational level (low, high), religion (religious, not religious), and ethnicity (Dutch, non-Dutch). Again, for all these analyses, stochastic uncertainty was dealt with using nonparametric bootstraps.

## Results

### Dropout and Sample Characteristics

**Figure 2:**
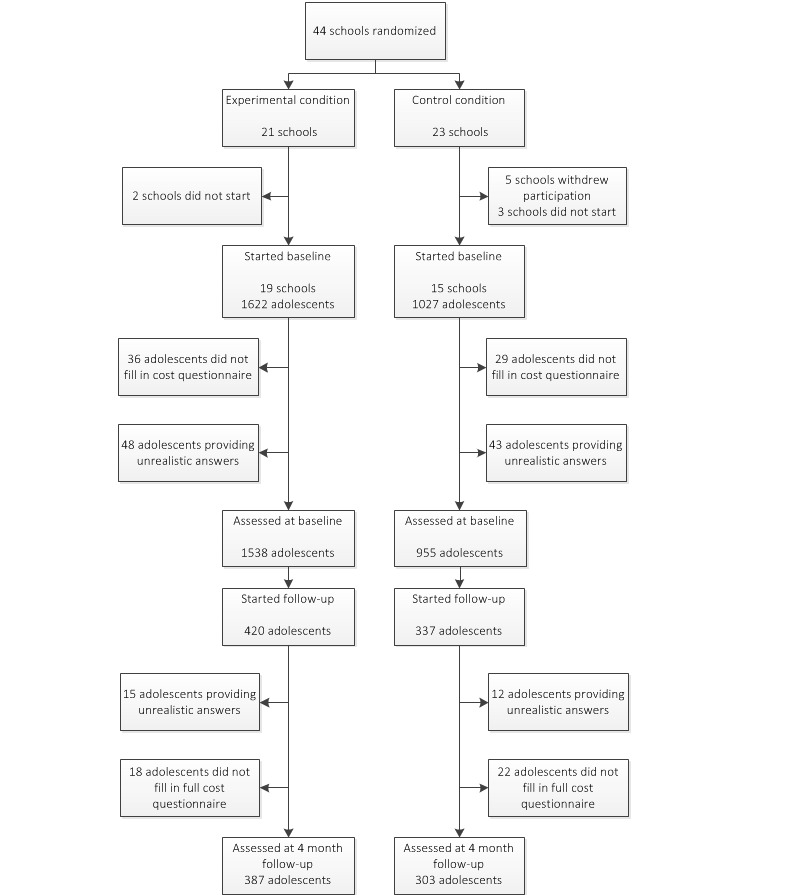
Flowchart describing the dropout of participants.

Figure 2shows a flowchart with the number of participating schools and adolescents at T0 and T1. In total, 44 schools were randomized into the control condition or intervention condition. Of the schools randomized to the control condition, five withdrew their participation before T0 (one secondary lower education, one lower vocational training, two secondary higher education, one secondary education mixed). In addition, three schools in the control condition (all secondary higher education) and two schools in the intervention condition (one lower vocational education, one higher secondary education) did not start the baseline assessment and did not respond to the emails and phone calls [ 24].

In total, 2649 adolescents from 34 schools participated in the baseline questionnaire. Of these, and different from the effect study [ 24], an additional 91 participants (3.4%) were excluded from analysis based on providing unrealistic answers to the cost questions. Another 65 participants were excluded from analysis because they did not answer the cost questions at T0. This resulted in 2493 adolescents who were included in the baseline analysis. Of these, 1538 were in the intervention condition and 955 were in the control condition ( Figure 2). The *t*tests and chi-square tests conducted on the baseline sample showed that the adolescents in the two conditions significantly differed on multiple characteristics. Adolescents in the intervention condition were significantly younger, more often female, had a higher educational level, more often indicated being religious, were less likely to be a drinker, were less often a binge drinker, had less binge drinking occasions, and had a lower weekly alcohol use than adolescents in the control condition ( Table 1).

Of the 2493 adolescents, 757 participated in the cost questionnaire at T1 (response rate 30.36%). Of these 757, another 27 participants (3.6%) were excluded from analysis based on having provided unrealistic answers to the cost questions. An additional 40 were excluded from analysis because they did not answer the cost questions at T1, resulting in 690 participants to be analyzed at baseline. Of these, 387 were in the intervention condition and 303 were in the control condition. Here, adolescents in the intervention condition were more often female, had a higher educational level, more often indicated being religious, and had a lower weekly alcohol use than participants in the control condition did ( Table 1).

For the T1 sample, results of the linear regression analyses show that weekly alcohol use was dependent on gender, age and educational level ( *R* ^*2*^=.146). For binge drinking occasions, a significant proportion of the variance could be explained by age and educational level ( *R* ^*2*^=.136).

**Table 1:** Baseline characteristics and differences at T0 and at T1.

Variable			Total (N=2493)	Intervention (n=1538)	Control (n=955)	Baseline difference (T0)			Follow-up difference (T1)		
						*t*_2492_	χ ^2^_1_	*P*	*t*_954_	χ ^2^_1_	*P*
Age (15-19 years), mean (SD)			16.3 (1.2)	16.0 (1.1)	16.7 (1.2)	15.08		<.001	1.78		.08
**Gender, n (%)**							45.6	<.001		16.5	<.001
	Male		1295 (51.95)	717 (46.62)	578 (60.5)						
	Female		1198 (48.05)	821 (53.38)	377 (39.5)						
**Educational level, n (%)**							73.4	<.001		6.7	.006
	High		1483 (59.49)	1017 (66.12)	466 (48.8)						
	Low		1010 (40.51)	521 (33.88)	489 (51.2)						
**Religion, n (%)**							10.0	.002		15.4	<.001
	No religion		1465 (58.76)	866 (56.31)	599 (62.7)						
	**Religion**		1028 (41.24)	672 (43.69)	356 (37.3)						
		Catholic	593 (23.79)	397 (25.81)	196 (20.5)						
		Protestant	174 (6.98)	130 (8.45)	44 (4.6)						
		Muslim	150 (6.02)	75 (4.88)	75 (7.9)						
		Other	111 (4.45)	70 (4.55)	41 (4.3)						
**Ethnicity, n (%)**							1.2	.27		0.0	.51
	Dutch		2225 (89.25)	1381 (89.79)	844 (88.4)						
	**Non-Dutch**		268 (10.75)	157 (10.21)	111 (11.6)						
		Antillean	5 (0.20)	3 (0.20)	2 (0.2)						
		Belgian	10 (0.40)	5 (0.33)	5 (0.5)						
		German	13 (0.52)	10 (0.65)	3 (0.3)						
		Surinamese	26 (1.04)	18 (1.17)	8 (0.8)						
		Moroccan	35 (1.40)	15 (0.98)	20 (2.1)						
		Turkish	48 (1.93)	21 (1.37)	27 (2.8)						
		Other	131 (5.25)	85 (5.53)	46 (4.8)						
**Alcohol use, n (%)**											
	Never drinkers		664 (26.63)	459 (29.84)	205 (21.5)		21.2	<.001		0.7	.39
	Binge drinkers		1271 (50.98)	724 (47.07)	547 (57.3)		24.5	<.001		3.6	.06
Binge drinking occasions, mean (SD)			2.2 (4.0)	2.0 (4.2)	2.4 (3.8)	2.51		.01	1.18		.24
Weekly alcohol use, mean (SD)			3.9 (8.8)	3.2 (8.1)	5.0 (9.6)	4.96		<.001	2.17		.03

### Costs

Table 2shows that the total health care costs per adolescent were lower in the intervention group (€85.65) than in the control group (€124.49). This difference can largely be explained by the difference in costs for reported hospital stays. However, costs of ICBs (€162.68) and substance use (€36.30) were higher in the intervention group than in the control group (€112.61 and €24.64, respectively). The difference in mean costs for court proceedings is noticeable and explains much of the difference in ICB-related costs. Large differences in specific costs were caused by outliers in either the intervention or the CAU arm. As indicated previously, sensitivity analyses were conducted without these outliers. When the intervention costs were included both from the health care and societal perspective, costs were higher in the intervention group. The *z*score for each cost category was positive and higher than the reference value of 1.96 [ 44], indicating that costs were skewed and tailed to the right ( Table 2).

**Table 2:** Mean and bootstrapped median costs per adolescent measured at T1 covering a 4-month period between T0 and T1 (€, 2014) and *z*scores per cost category.

Type of costs		Intervention group (€)		Control group (€)		Skewness (z score) ^b^
		Mean (SD)	Median (σ ^2^) ^a^	Mean (SD)	Median (σ ^2^) ^a^	
Intervention costs		53.00 (0.00)	53.00 (0.00)			
**Health care costs**						
	General practitioner	25.42 (3.39)	25.22 (11.49)	23.84 (4.36)	23.50 (11.49)	73.44
	Emergency care	10.16 (3.14)	10.06 (9.86)	8.08 (3.74)	7.65 (13.99)	97.98
	Hospital stays	27.22 (12.38)	26.88 (135.26)	67.90 (58.94)	66.98 (3473.92)	258.45
	Ambulance rides	7.34 (2.95)	6.74 (8.70)	9.37 (6.18)	8.64 (38.19)	174.83
	Mental health care	16.07 (6.37)	15.59 (40.58)	14.16 (5.72)	13.78 (32.71)	115.83
	Total health care costs	85.65 (20.62)	84.89 (425.18)	124.49 (67.91)	120.38 (4611.76)	229.86
Total health care perspective		139.16 (20.77)	138.04 (431.39)	127.45 (68.64)	122.12 (4711.45)	229.09
**Educational sector costs**						
	School absenteeism	51.88 (7.13)	51.44 (50.84)	66.77 (11.03)	65.74 (121.66)	76.31
	Attendance officer	0.03 (0.03)	0.04 (0.00)	2.53 (2.16)	2.40 (4.67)	272.90
	Total educational sector costs	51.52 (7.16)	51.17 (51.27)	69.30 (11.35)	68.37 (128.82)	74.60
**Labor and social security costs**						
	Work absenteeism	9.67 (3.49)	9.20 (12.18)	6.03 (1.41)	6.01 (1.99)	140.85
	Total labor and social security costs	9.63 (3.39)	9.30 (11.49)	6.09 (1.40)	6.01 (1.96)	140.85
**Household and leisure costs**						
	Failure to perform household activities	8.58 (3.01)	8.31 (9.06)	10.92 (3.39)	10.74 (11.49)	98.06
	Failure to perform other activities	25.77 (13.20)	24.67 (174.24)	8.17 (1.60)	8.02 (2.56)	182.19
	Youth and family center	0.00 (0.00)	0.00 (0.00)	0.33 (0.34)	0.33 (0.12)	282.45
	Family care	1.26 (1.27)	1.49 (1.61)	0.18 (0.18)	0.19 (0.03)	270.73
	Total household and leisure costs	35.38 (14.80)	33.55 (219.04)	19.23 (4.21)	18.81 (17.72)	163.43
**Criminal justice system costs**						
	Police services	4.68 (2.61)	4.45 (6.81)	6.67 (2.50)	6.47 (6.25)	126.44
	Youth police services	1.58 (1.72)	1.91 (2.96)	0.28 (0.20)	0.28 (0.04)	277.89
	Court proceedings	55.10 (34.45)	50.92 (1186.80)	9.95 (7.39)	10.15 (54.61)	197.40
	Child protection services	0.29 (0.31)	0.36 (0.10)	0.68 (0.72)	0.72 (0.52)	227.11
	Child health protection services	3.13 (3.73)	4.06 (13.91)	0.46 (0.45)	0.48 (0.20)	277.12
	Total criminal justice system costs	66.32 (38.43)	61.51 (1476.86)	18.77 (9.79)	17.40 (95.84)	182.55
**Total intersectoral costs**		162.68 (41.85)	158.61 (1751.42)	112.61 (18.83)	111.26 (354.57)	124.84
Substance use costs						
	Cigarettes	30.68 (6.25)	30.46 (39.06)	18.82 (3.94)	18.82 (15.52)	48.86
	Soft drugs	4.30 (1.95)	4.00 (3.80)	5.44 (3.01)	5.12 (9.06)	153.72
	Hard drugs	1.15 (1.08)	1.32 (1.17)	0.20 (0.11)	0.19 (0.01)	275.97
	Total costs of substance use	36.30 (7.58)	36.09 (57.46)	24.64 (5.85)	24.30 (34.22)	61.42
Total societal perspective		336.45 (53.31)	331.41 (2841.96)	263.52 (70.70)	255.75 (4998.49)	135.60

^a^The presented median cost is the 50 ^th^percentile of 1000 bootstrap replications.

^b^The *z*score for each cost category is positive and higher than the reference value of 1.96 [ 44] indicating that costs were skewed and tailed to the right.

### Incremental Costs

Table 3shows costs per condition. Note that these means slightly differ from the costs presented in Table 2, for costs in Table 2are bootstrapped means, whereas costs in Table 3are means drawn from raw data. For both perspectives, costs were higher in the intervention condition. The incremental costs (ie, the difference in mean costs per adolescent between the intervention and control condition) varied per perspective, namely €13.76 from the health care perspective and €74.03 from the societal perspective.

**Table 3:** Summary statistics for the base case sensitivity cost-effectiveness bootstrap analyses.

Perspective ^a^and condition				Costs (€) ^b^	Effect ^c^	ICER ^d^	NE	NW (inferior)	SW	SW (dominant)
**Base case analyses**										
	**Weekly alcohol use**									
		**Health care**								
			Control (n=303)	125.32	–1.51					
			Intervention (n=387)	139.08	–0.78	40	55%	10%	6%	30%
		**Societal**								
			Control (n=303)	262.68	–1.51					
			Intervention (n=387)	336.71	–0.78	62	60%	14%	3%	23%
	**Binge drinking occasions**									
		**Health care**								
			Control (n=303)	125.32	–0.33					
			Intervention (n=387)	139.08	0.16	79	60%	4%	2%	34%
		**Societal**								
			Control (n=303)	262.68	–0.33					
			Intervention (n=387)	336.71	0.16	144	69%	5%	1%	25%
**Sensitivity analyses excluding outliers**										
	**Weekly alcohol use**									
		**Health care**								
			Control (n=302)	59.47	–1.55					
			Intervention (n=387)	139.08	–0.78	72	82%	17%	0%	1%
		**Societal**								
			Control (n=302)	193.85	–1.55					
			Intervention (n=384)	269.19	–0.66	67	80%	12%	1%	7%
	**Binge drinking occasions**									
		**Health care**								
			Control (n=302)	59.47	–0.33					
			Intervention (n=387)	139.08	0.16	140	93%	6%	0%	1%
		**Societal**								
			Control (n=302)	193.85	–0.33					
			Intervention (n=384)	269.19	0.21	124	87%	4%	0%	9%

^a^Bootstrap analyses were conducted from two perspectives: the health care perspective and the societal perspective.

^b^Mean costs per adolescent at 2014 prices.

^c^Reduction in per week alcohol use or binge drinking occasions between T0 and T1, with negative values indicating an increase at T1 compared to T0.

^d^The presented ICER is the 50 ^th^percentile of 5000 bootstrap replications of the ICER.

### Incremental Effects

Table 3shows the effects per condition. In comparison with the control condition, the intervention was incrementally effective in reducing the weekly use of alcohol and number of binge drinking occasions. At T1, adolescents in the control condition drank a mean 1.51 glasses of alcohol per week more than at T0. In the intervention condition, there was an increase of 0.78 glasses, resulting in a mean incremental effect of 0.73 glasses per week. Furthermore, in the control condition, there was an increase of 0.33 binge drinking occasions. In the intervention condition, there was a decrease of 0.16, resulting in a mean incremental effect of 0.49 binge drinking occasions per 30 days. For both outcome measures, this did not change with perspective; a change of perspective within the base case scenario stipulated only a change in costs.

#### Incremental Cost-Effectiveness Ratios

From both perspectives, the mean costs were higher for the intervention condition in comparison with the control condition. Since the intervention was more effective than CAU on both outcome measures, this resulted in positive ICERs ( Table 3). However, ICERs differed for both perspectives, namely €40 and €79 from the health care perspective, and €62 and €144 from the societal perspective per incremental reduction of one glass of alcohol per week and binge drinking occasion per 30 days, respectively.

**Figure 3:**
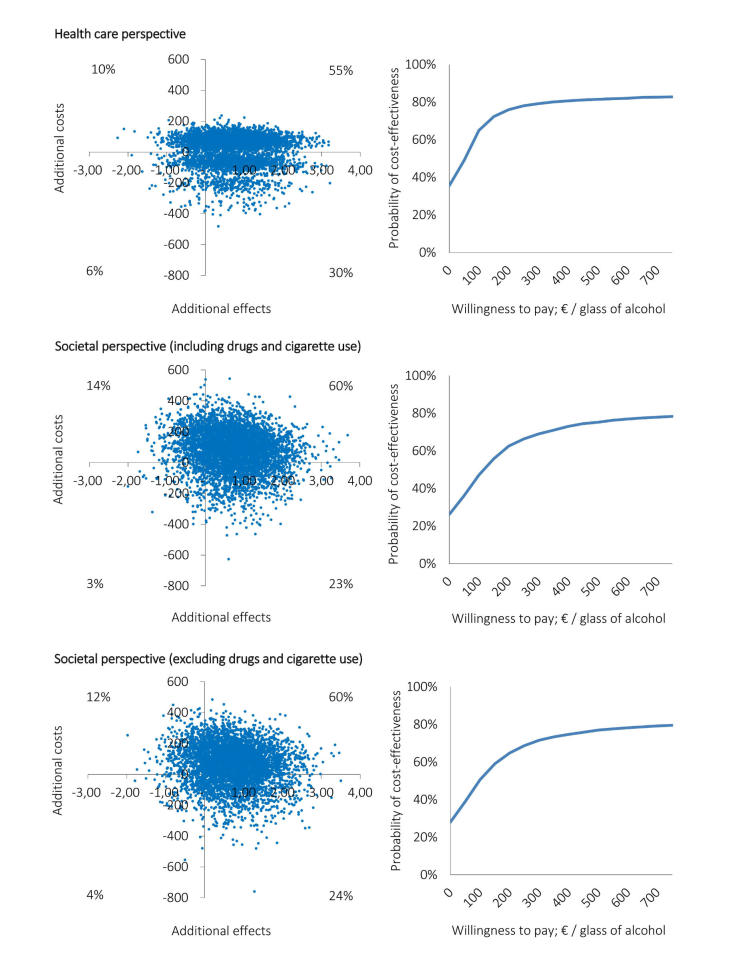
Cost-effectiveness planes (left side) and corresponding CEACs (right side) of the economic evaluations based on the weekly alcohol use outcome measure, which were conducted from the health care perspective (upper), societal perspective including drugs and cigarette use (middle), and societal perspective excluding drugs and cigarette use (lower).

**Figure 4:**
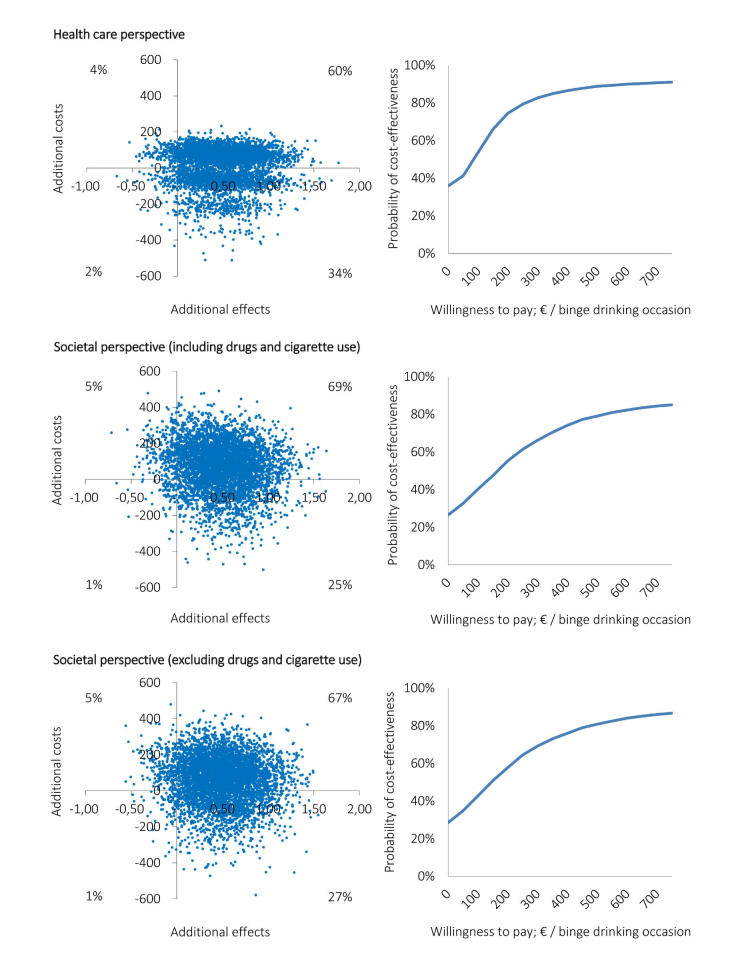
Cost-effectiveness planes (left side) and corresponding CEACs (right side) of the economic evaluations based on the binge drinking occasions outcome measure, which were conducted from the health care perspective (upper), societal perspective including drugs and cigarette use (middle), and societal perspective excluding drugs and cigarette use (lower).

The cost-effectiveness planes ( Figures 3and 4, left side) show differences in distributions of the 5000 simulated ICERs across the four quadrants between the CEAs carried out from the two perspectives. Corresponding with the median ICERs presented in Table 3, the majority of simulated ICERs for all base case analyses are located in the NE quadrant. However, the distribution of the simulated ICERs among the quadrants differs between the perspectives. Notable is the shift of the cloud of ICERs toward the SE quadrant in the analysis carried out from the health care perspective (ie, 30% for weekly alcohol use and 34% for binge drinking occasions) in comparison to the analyses carried out from the societal perspective (ie, 23% and 25%, respectively).

The preceding percentages equal the probabilities of the intervention being cost-effective at a WTP max of €0 in the CEACs ( Figures 3and 4, right side). These results show that for low WTP thresholds the probability of the intervention being cost-effective over the control intervention is higher from a health care perspective than it is from the societal perspective. For all base case analyses, the vast majority of simulated incremental effects were in the NE quadrant; therefore, these probabilities increase to approximately 80% when the WTP max increases. The probabilities of the intervention being cost-effective do not differ much between the two perspectives for WTP thresholds greater than €500 ( Figures 3and 4, right side).

### Sensitivity and Subgroup Analyses

The results of the sensitivity analyses (ie, excluding cost outliers) attest to the robustness of the base case analyses ( Table 3). From the societal perspective, ICERs were close to similar. From the health care perspective, ICERs increased and were higher than those of the societal perspective. However, as for the base case analyses, the probability of the intervention being cost-effective remained dependent on the WTP max. The results of the analyses conducted from a societal perspective minus the costs of drugs and cigarette use were similar to the results of the analyses conducted in which these costs were included ( Figures 3and 4).

Subgroup analyses showed, from both the health care and the societal perspective, and for both outcome measures, that the intervention was cost-effective for the older adolescents and those at a lower educational level ( Tables 4and 5). From a health care perspective, it was found to be cost-effective for the male and nonreligious adolescent subgroups as well. The intervention was not cost-effective for those with a non-Dutch ethnicity or for female adolescents for the weekly alcohol use outcome measure. For all other subgroups, ICERs were positive, meaning the intervention was cost-effective depending on the WTP max. The corresponding cost-effectiveness planes and CEACs of all subgroup analyses can be obtained in Multimedia Appendices 2and 3.

**Table 4:** Summary statistics for the subgroup sensitivity cost-effectiveness bootstrap analyses based on the weekly alcohol use outcome measure.

Perspective ^a^and condition				Costs (€) ^b^	Effect ^c^	ICER ^d^	NE	NW (inferior)	SW	SE (dominant)
**Gender subgroups**										
	**Male**									
		**Health care**								
			Control (n=162)	193.96	–2.19					
			Intervention (n=147)	164.53	–0.80	Dominant	43%	2%	3%	52%
		**Societal**							
			Control (n=162)	337.21	–2.19					
			Intervention (n=147)	352.43	–0.80	21	56%	3%	2%	39%
	**Female**									
		**Health care**								
			Control (n=141)	46.46	–0.73					
			Intervention (n=240)	123.48	–0.77	Inferior	48%	52%	0%	0%
		**Societal**								
			Control (n=141)	177.04	–0.73					
			Intervention (n=240)	327.09	–0.77	Inferior	46%	52%	1%	1%
**Age subgroups**										
	**Younger adolescents (15-16 years)**									
		**Health care**								
			Control (n=200)	50.05	–1.24					
			Intervention (n=281)	145.79	–0.73	108	80%	20%	0%	0%
		**Societal**								
			Control (n=200)	177.07	–1.24					
			Intervention (n=281)	334.90	–0.73	149	77%	20%	0%	3%
	**Older adolescents (≥17 years)**									
		**Health care**								
			Control (n=103)	271.48	–2.03					
			Intervention (n=106)	121.27	–0.92	Dominant	28%	5%	11%	56%
		**Societal**								
			Control (n=103)	428.90	–2.03					
			Intervention (n=106)	341.53	–0.92	Dominant	32%	7%	10%	51%
**Educational level subgroups**										
	**Low**									
		**Health care**								
			Control (n=98)	263.02	–2.20					
			Intervention (n=91)	117.35	–0.60	Dominant	31%	4%	7%	58%
		**Societal**								
			Control (n=98)	435.78	–2.20					
			Intervention (n=91)	282.38	–0.60	Dominant	26%	4%	8%	63%
	**High**									
		**Health care**								
			Control (n=205)	59.50	–1.18					
			Intervention (n=296)	145.76	–0.83	102	73%	26%	0%	1%
		**Societal**								
			Control (n=205)	179.93	–1.18					
			Intervention (n=296)	353.42	–0.83	172	70%	28%	0%	1%
**Religion subgroups**										
	**Religion**									
		**Health care**								
			Control (n=97)	46.91	–2.05					
			Intervention (n=181)	148.11	–0.71	66	92%	8%	0%	0%
		**Societal**								
			Control (n=97)	155.80	–2.05					
			Intervention (n=181)	336.35	–0.71	110	90%	9%	0%	1%
	**No religion**									
		**Health care**								
			Control (n=206)	162.24	–1.25					
			Intervention (n=206)	131.13	–0.84	Dominant	37%	14%	13%	37%
		**Societal**								
			Control (n=206)	313.00	–1.25					
			Intervention (n=206)	337.04	–0.84	5	42%	19%	8%	31%
**Ethnicity subgroups**										
	**Dutch**									
		**Health care**								
			Control (n=278)	128.83	–1.65					
			Intervention (n=356)	136.90	–0.84	36	53%	8%	5%	34%
		**Societal**								
			Control (n=278)	262.96	–1.65					
			Intervention (n=356)	334.67	–0.84	57	60%	11%	3%	26%
	**Other**									
		**Health care**								
			Control (n=25)	86.35	0.08					
			Intervention (n=31)	164.09	–0.10	Inferior	32%	51%	4%	13%
		**Societal**								
			Control (n=25)	259.53	0.08					
			Intervention (n=31)	360.20	–0.10	Inferior	23%	49%	7%	21%

^a^Bootstrap analyses were conducted from two perspectives: the health care perspective and the societal perspective.

^b^Costs per adolescent at 2014 prices.

^c^Reduction in per week alcohol use between T0 and T1, with negative values indicating an increase at T1 compared to T0.

^d^The presented ICER is the 50 ^th^percentile of 5000 bootstrap replications of the ICER.When an ICER is negative, then it is labeled as being either “dominant” (suggesting that the intervention is both more effective and less costly than CAU) or “inferior” (suggesting that the intervention is both less effective and more costly than CAU).

**Table 5:** Summary statistics for the subgroup sensitivity cost-effectiveness bootstrap analyses based on the binge drinking occasions outcome measure.

Perspective ^a^and condition				Costs (€) ^b^	Effect ^c^	ICER ^d^	NE	NW (inferior)	SW	SE (dominant)
**Gender subgroups**										
	**Male**											
		**Health care**							
			Control (n=162)	193.96	–0.57					
			Intervention (n=147)	164.53	0.08	Dominant	44%	1%	1%	54%
		**Societal**							
			Control (n=162)	337.21	–0.57					
			Intervention (n=147)	352.43	0.08	46	57%	1%	1%	42%
	**Female**									
		**Health care**							
			Control (n=141)	46.46	–0.04					
			Intervention (n=240)	123.48	0.21	179	81%	19%	0%	0%
		**Societal**							
			Control (n=141)	177.04	–0.04					
			Intervention (n=240)	327.09	0.21	291	78%	19%	0%	2%
**Age subgroups**										
	**Younger adolescents (15-16 years)**									
		**Health care**								
			Control (n=200)	50.05	–0.26					
			Intervention (n=281)	145.79	0.13	276	71%	29%	0%	0%
		**Societal**								
			Control (n=200)	177.07	–0.26					
			Intervention (n=281)	334.90	0.13	343	68%	29%	1%	2%
	**Older adolescents (≥17 years)**									
		**Health care**								
			Control (n=103)	271.48	–0.45					
			Intervention (n=106)	121.27	0.94	Dominant	31%	0%	0%	68%
		**Societal**								
			Control (n=103)	428.90	–0.45					
			Intervention (n=106)	341.53	0.94	Dominant	39%	0%	0%	60%
**Educational level subgroups**										
	**Low**										
		**Health care**								
			Control (n=98)	263.02	–0.53					
			Intervention (n=91)	117.35	1.04	Dominant	35%	0%	0%	64%
		**Societal**								
			Control (n=98)	435.78	–0.53					
			Intervention (n=91)	282.38	1.04	Dominant	30%	0%	0%	70%
	**High**									
		**Health care**								
			Control (n=205)	59.50	–0.23					
			Intervention (n=296)	145.76	–0.10	231	69%	30%	0%	1%
		**Societal**								
			Control (n=205)	179.93	–0.23					
			Intervention (n=296)	353.42	–0.10	435	69%	29%	0%	1%
**Religion subgroups**										
	**Religion**									
		**Health care**								
			Control (n=97)	46.91	–0.34					
			Intervention (n=181)	148.11	0.32	148	96%	4%	0%	0%
		**Societal**								
			Control (n=97)	155.80	–0.34					
			Intervention (n=181)	336.35	0.32	256	95%	4%	0%	1%
	**No religion**									
		**Health care**								
			Control (n=206)	162.24		–0.32					
			Intervention (n=206)	131.13	0.02	Dominant	43%	7%	6%	45%
		**Societal**								
			Control (n=206)	313.00	–0.32					
			Intervention (n=206)	337.04	0.02	47	50%	8%	4%	37%
**Ethnicity subgroups**											
	**Dutch**									
		**Health care**								
			Control (n=278)	128.83	–0.36					
			Intervention (n=356)	136.90	0.18	71	59%	3%	2%	37%
		**Societal**								
			Control (n=278)	262.96	–0.36					
			Intervention (n=356)	334.67	0.18	139	69%	4%	1%	26%
	**Other**									
		**Health care**								
			Control (n=25)	86.35	0.00					
			Intervention (n=31)	164.09	0.03	Inferior	36%	48%	3%	13%
		**Societal**								
			Control (n=25)	259.53	0.00					
			Intervention (n=31)	360.20	0.03	Inferior	24%	48%	4%	24%

^a^Bootstrap analyses were conducted from two perspectives: the health care perspective and the societal perspective.

^b^Costs per adolescent at 2014 prices.

^c^Reduction in per week alcohol use between T0 and T1, with negative values indicating an increase at T1 compared to T0.

^d^The presented ICER is the 50 ^th^percentile of 5000 bootstrap replications of the ICER.When an ICER is negative, then it is labeled as being either “dominant” (suggesting that the intervention is both more effective and less costly than CAU) or “inferior” (suggesting that the intervention is both less effective and more costly than CAU).

## Discussion

### Principal Results

To the best of our knowledge, this was the first cost-effectiveness analysis of a Web-based intervention conducted from both the health care and societal perspective that also incorporated the possible impact of ICBs on the cost-effectiveness results. From both a health care and a societal perspective, our study shows the intervention was incrementally more effective and more costly than CAU. This counts for both the analyses in which the weekly alcohol use outcome measure was incorporated and the analyses based on the binge drinking occasions outcome measure.

Although the intervention was incrementally effective in targeting weekly alcohol use, there was an increase in the number of glasses of alcohol between T0 and T1 in both arms of the trial. This can be explained based on the estimation that approximately one-third of the study sample had his or her birthday during this 4-month period between T0 and T1, of which some reached the legal drinking age of 18 years. Furthermore, all adolescents in the sample aged 4 months, which increased the chance of them starting to drink or drink more. This is also true for the younger Dutch adolescents because many start drinking before the legal drinking age [ 45]. However, contrary to weekly alcohol use, the number of binge drinking occasions did not increase in the intervention arm; a small decrease of a mean 0.16 binge drinking occasions was noticed compared to an increase of a mean 0.33 in the CAU arm. Therefore, relative to the overall alcohol intake and compared to CAU, it can tentatively be concluded that adolescents in the intervention arm became less irresponsible about drinking.

Our research also shows that the inclusion of ICBs in the economic evaluation impacted the cost-effectiveness results of the analysis, especially for certain subgroups. From a health care perspective, the intervention is cost-effective for the male, lower education, older adolescent, and nonreligious subgroups. However, from a societal perspective (which includes ICBs), the intervention is clearly cost-effective only for the lower education and older adolescent subgroups.

The inferiority of the intervention for certain subgroups could, among other reasons, partly be explained based on the finding that the baseline consumption for these subgroups was relatively low compared to that of their counterparts. For example, the baseline mean weekly alcohol use in the female subgroup was 1.49 glasses (SD 3.57) compared to weekly mean 4.18 glasses (SD 8.42) in the male subgroup. In so far as the following can be concluded based on an analysis of the smallest subgroup (n=56), this also goes for the non-Dutch subgroup (mean 2.16, SD 5.00 glasses and mean 1.21, SD 2.44 binge drinking occasions) versus the Dutch subgroup (mean 2.74, SD 6.48 glasses and mean 1.64, SD 2.77 binge drinking occasions). In these subgroups, there was less effect to be gained. Consequently, this could be related to the possibility of these adolescents not identifying themselves as being part of the target group of, and being affected by, the intervention.

### Strengths and Limitations

Some of the strengths of this study are its relatively large sample size and its randomized design. The cluster randomization at the school level minimized the risk of contamination between the study conditions. Furthermore, the large heterogenic study sample was a good representation of the Dutch adolescent school-going population and allowed for subgroup analyses on multiple background variables.

The use of a societal perspective along with a health care perspective was a major strength of this study. The societal perspective is argued to be dominant over other perspectives [ 46- 48]. This is because of, but not restricted to, health economics’ foundations in welfare economics, which means that an economic evaluation should include the impact of an intervention on the whole society [ 46]. However, not only the choice of perspective, but also the way this was implemented in the study design, can be considered a major strength. Because the study population consisted of school-going adolescents, limiting the societal perspective to including merely labor productivity costs would not have properly reflected this impact. For this study, the results show that labor productivity costs make up just a small part of the total costs of ICBs ( Table 2). By including costs within the educational sector and criminal justice system, we managed to provide a better reflection of the economic impact of this intervention on society.

Apart from these strengths, the findings of this study need to be placed in the context of the study’s limitations. First, both at T0 and T1, the composition of adolescents in the intervention condition was significantly different from that of the control condition for various characteristics, including gender, educational level, and religion. This might have been caused by (1) the cluster RCT design instead of randomization at the individual level and/or (2) the large dropout before the baseline assessment within the control condition in comparison with the intervention condition. Although the results of the regression analysis showed a relationship between some of the background variables and the outcome measures, uncertainty around the ICERs that were calculated in the base case analyses was dealt with through various strategies. The sensitivity analyses attest to the robustness of the findings. Furthermore, the heterogeneity of the sample was addressed extensively by calculating ICERs and conducting bootstrap analyses for all subgroups based on all background variables.

Second, the follow-up period of this CEA (ie, 4 months) might be regarded as short. Costs and (health) benefits that fall beyond these 4 months were not assessed. Future studies, including additional follow-up measures and cost-effectiveness modeling, could be interesting. Other studies have shown that the cumulative cost savings in the life span of health promotion interventions for adolescents could be high [ 49].

Third, the authors decided not to further modify the original dataset by imputation and restricted the analysis to complete cases. A missing completely at random analysis (MCAR) in SPSS version 20 based on the N=2493 sample that started at baseline showed 71% to 72% per cost variable at T1. Furthermore, the missing values were not at random ( *P*<.001). Although this was expected considering the large dropout between T0 and T1, the same goes for the n=757 sample that started follow-up ( *P*<.001). Given the nonnormality of cost variables, the nonrandomness of missing values, and the large dropout as is common in many Web-based interventions [ 19, 50- 55], it was concluded that additional imputation would have manipulated the original dataset too much. This counts for both the basic imputation methods, such as expectation maximization and last observation carried forward, as well as for the more advanced methods, such as Markov chain Monte Carlo technique with predictive mean matching [ 56]. As for imputation strategies, the chosen strategy might have led to biased results. Nevertheless, the alternative of replacing more than 70% of the values, which would have been needed in this study, would have increased the risk of a type II error [ 51]. Imputation would have resulted in an increased chance of underestimating the intervention’s effectiveness and an unrealistic representation of costs.

Fourth, measurements were based on self-reports, which could have led to an underestimation of service use, alcohol use, and use of other goods in comparison with daily diaries [ 24, 57]. As for any measurement based on recalling services or goods used, this is because of forgetting [ 17]. However, in this study, recall periods were kept short. For example, respondents were asked for their alcohol use in the previous week and not in a typical week. Furthermore, the recall period for the cost measurement questions was only 4 months. In addition, because the groups were randomized, this underestimation is likely to be equally distributed among the intervention and control group. Therefore, it is unlikely that the ICERs were affected.

Finally, within the setting of this study, respondents were free to fill in the answers themselves during the measurements. As mentioned earlier, limits needed to be set to exclude respondents who provided unrealistic answers. The choice made by the authors to exclude whole cases might have affected the outcomes of the analysis. However, this choice is justifiable based on the finding that the vast majority of unrealistic answers were not even close to the limits set by the authors and were far higher than the credible range. For example, there were seven respondents who claimed to have spent more than 200 nights in a hospital bed in the previous 4 months, of which three said to have spent more than 200,000. The 27 adolescents at T1 who filled in unrealistic answers (only 3.6% of the n=757 sample that started follow-up) had a mean 2.9 unrealistic answers. Based on the data, it was clear that the vast majority of these 27 adolescents did this deliberately and systematically. Although the outcome of the analyses might have been affected by the limits that were set by the authors, these limits were carefully considered, discussed, and decided a priori to the analyses. This was done to minimize the chance of biased results. Furthermore, the impact of cost outliers on the outcomes of the base case analyses has been covered in sensitivity analyses in which cost outliers were excluded.

### Recommendations

Computer-tailored feedback can be a cost-effective way to target alcohol use and binge drinking among adolescents. In the Netherlands, despite the Dutch government’s change of policy to reduce the minimum legal drinking age, 33.4% of Dutch adolescents were drinkers in 2014 [ 45]. This is because in practice drinking rules are set not only by Dutch law, but also by parents or caregivers and alcoholic beverages can easily be obtained via family and friends. Therefore, effective and cost-effective interventions targeting adolescent drinking behavior are still very much needed. The high dissemination capabilities of the Alcoholic Alert intervention, combined with its solid basis in the I-Change model and low intervention costs could make it an interesting investment for reducing alcohol use among adolescents.

Because the cost-effectiveness for the whole sample is dependent on the WTP max per effect, it is difficult to make strong recommendations on whether the intervention should be implemented from an economic point of view. Contrary to the generic outcome measure quality-adjusted life year (QALY) [ 58, 59], and as is common for the majority of specific outcome measures, no guidelines are available that provide a reference cost-effectiveness threshold for reducing the consumption of alcohol. However, the CEACs provide decision supportive information because these provide cost-effectiveness probabilities for a wide range of hypothetical thresholds for all analyses. These also show that, from both the health care and the societal perspective, the intervention is cost-effective for older adolescents and for those at a lower educational level, regardless of which threshold is set. From a health economic viewpoint, it is recommended that these specific groups be targeted. When adopting a health care perspective, the same goes for the male and nonreligious adolescent subgroups.

In general, policy makers should be aware of the impact of the perspective chosen for the analysis on its outcomes. Omitting ICBs could negatively affect the reliability and informative value of analyses that are conducted from a societal perspective. Therefore, it is recommended that researchers should carefully make a priori considerations on the costs to be included because leaving out important costs could lead to biased results [ 60]. Finally, as in this study, high attrition rates could affect the outcomes of CEAs. High attrition rates are common in eHealth interventions [ 61, 62]. It is recommended that more research should be conducted on adherence to eHealth interventions and that these interventions be implemented in practice, thus increasing their effectiveness, cost-effectiveness, and impact on public health.

## Appendix

Multimedia Appendix 1 Additional File 1 List of limits of realistic answers to the questions in the cost questionnaires at T0 and T1.

Multimedia Appendix 2 Additional File 2 Cost-effectiveness planes (left side) and corresponding CEACs (right side) of the economic evaluations based on the weekly alcohol use outcome measure, which were conducted from the health care perspective (upper), societal perspective including drugs and cigarette use (middle) and societal perspective excluding drugs and cigarette use (below) for the subgroups: male/female, younger/older, lower education/higher education, religious/non-religious, and Dutch/non-Dutch.

Multimedia Appendix 3 Additional File 3 Cost-effectiveness planes (left side) and corresponding CEACs (right side) of the economic evaluations based on binge drinking occasions outcome measure, which were conducted from the health care perspective (upper), societal perspective including drugs and cigarette use (middle) and societal perspective excluding drugs and cigarette use (below) for the subgroups: male/female, younger/older, lower education/higher education, religious/non-religious, and Dutch/non-Dutch.

## Abbreviations

CAU: care as usual
CEA: cost-effectiveness analysis
CEAC: cost-effectiveness acceptability curve
ICB: intersectoral costs and benefit
ICC: intracluster correlation coefficient
ICER: incremental cost-effectiveness ratio
MCAR: missing completely at random
NE: northeast quadrant
NW: northwest quadrant
RCT: randomized controlled trial
SE: southeast quadrant
SW: southwest quadrant
WTP max: maximum willingness to pay

## References

[1] Rehm J, Taylor B, Room R (2006). Global burden of disease from alcohol, illicit drugs and tobacco. Drug Alcohol Rev.

[2] Ezzati M, Lopez A, Rodgers A, Murray C (2004). World Health Organization.

[3] Smit F, Lokkerbol J, Riper H, Majo MC, Boon B, Blankers M (2011). Modeling the cost-effectiveness of health care systems for alcohol use disorders: how implementation of eHealth interventions improves cost-effectiveness. J Med Internet Res.

[4] (2014). World Health Organization.

[5] Mohapatra S, Patra J, Popova S, Duhig A, Rehm J (2010). Social cost of heavy drinking and alcohol dependence in high-income countries. Int J Public Health.

[6] Rehm J, Mathers C, Popova S, Thavorncharoensap M, Teerawattananon Y, Patra J (2009). Global burden of disease and injury and economic cost attributable to alcohol use and alcohol-use disorders. Lancet.

[7] Smit F, Cuijpers P, Oostenbrink J, Batelaan N, de GR, Beekman A (2006). Costs of nine common mental disorders: implications for curative and preventive psychiatry. J Ment Health Policy Econ.

[8] (2002). World Health Organization.

[9] (2006). Youth Violence and Alcohol.

[10] Chiauzzi E, Green TC, Lord S, Thum C, Goldstein M (2005). My student body: a high-risk drinking prevention web site for college students. J Am Coll Health.

[11] Kypri K, Saunders JB, Williams SM, McGee RO, Langley JD, Cashell-Smith ML, Gallagher SJ (2004). Web-based screening and brief intervention for hazardous drinking: a double-blind randomized controlled trial. Addiction.

[12] Lustria ML, Cortese J, Noar SM, Glueckauf RL (2009). Computer-tailored health interventions delivered over the Web: review and analysis of key components. Patient Educ Couns.

[13] Matano RA, Koopman C, Wanat SF, Winzelberg AJ, Whitsell SD, Westrup D, Futa K, Clayton JB, Mussman L, Taylor CB (2007). A pilot study of an interactive web site in the workplace for reducing alcohol consumption. J Subst Abuse Treat.

[14] Riper H, Kramer J, Smit F, Conijn B, Schippers G, Cuijpers P (2008). Web-based self-help for problem drinkers: a pragmatic randomized trial. Addiction.

[15] van der Wulp NY, Hoving C, Eijmael K, Candel MJ, van Dalen W, De Vries H (2014). Reducing alcohol use during pregnancy via health counseling by midwives and internet-based computer-tailored feedback: a cluster randomized trial. J Med Internet Res.

[16] de Vries H, Brug J (1999). Computer-tailored interventions motivating people to adopt health promoting behaviours: introduction to a new approach. Patient Educ Couns.

[17] Lemmens P, Tan ES, Knibbe RA (1992). Measuring quantity and frequency of drinking in a general population survey: a comparison of five indices. J Stud Alcohol.

[18] van Keulen HM, Mesters I, Brug J, Ausems M, Campbell M, Resnicow K, Zwietering PJ, van Breukelen G, van Mechelen W, Severens JL, de Vries H (2008). Vitalum study design: RCT evaluating the efficacy of tailored print communication and telephone motivational interviewing on multiple health behaviors. BMC Public Health.

[19] Smit ES, Evers SM, de Vries H, Hoving C (2013). Cost-effectiveness and cost-utility of Internet-based computer tailoring for smoking cessation. J Med Internet Res.

[20] Kreuter MW, Wray RJ (2003). Tailored and targeted health communication: strategies for enhancing information relevance. Am J Health Behav.

[21] Schulz DN, Smit ES, Stanczyk NE, Kremers SP, de Vries H, Evers SM (2014). Economic evaluation of a web-based tailored lifestyle intervention for adults: findings regarding cost-effectiveness and cost-utility from a randomized controlled trial. J Med Internet Res.

[22] (2014). Internet World Stats.

[23] (2015). Eurostat.

[24] Jander A, Crutzen R, Mercken L, Candel M, de Vries H (2016). Effects of a web-based computer-tailored game to reduce binge drinking among dutch adolescents: a cluster randomized controlled trial. J Med Internet Res.

[25] Voogt CV, Kleinjan M, Poelen EA, Lemmers LA, Engels RC (2013). The effectiveness of a web-based brief alcohol intervention in reducing heavy drinking among adolescents aged 15-20 years with a low educational background: a two-arm parallel group cluster randomized controlled trial. BMC Public Health.

[26] Suijkerbuijk A, Van Gils P, De Wit G (2014). Bilthoven: National Institute for Public Health and the Environment.

[27] Blankers M, Nabitz U, Smit F, Koeter MW, Schippers GM (2012). Economic evaluation of internet-based interventions for harmful alcohol use alongside a pragmatic randomized controlled trial. J Med Internet Res.

[28] Ingels JB, Corso PS, Kogan SM, Brody GH (2013). Cost-effectiveness of the strong African American families-teen program: 1-year follow-up. Drug Alcohol Depend.

[29] Nelson JP (2015). Binge drinking and alcohol prices: a systematic review of age-related results from econometric studies, natural experiments and field studies. Health Econ Rev.

[30] Drost RM, Paulus AT, Ruwaard D, Evers SM (2013). Inter-sectoral costs and benefits of mental health prevention: towards a new classification scheme. J Ment Health Policy Econ.

[31] Jander A, Crutzen R, Mercken L, de Vries H (2014). A Web-based computer-tailored game to reduce binge drinking among 16 to 18 year old Dutch adolescents: development and study protocol. BMC Public Health.

[32] Wechsler H, Dowdall GW, Davenport A, Rimm EB (1995). A gender-specific measure of binge drinking among college students. Am J Public Health.

[33] de Vries H, Mudde A, Leijs I, Charlton A, Vartiainen E, Buijs G, Clemente MP, Storm H, González NA, Nebot M, Prins T, Kremers S (2003). The European Smoking Prevention Framework Approach (EFSA): an example of integral prevention. Health Educ Res.

[34] Johannesson M (1997). Avoiding double-counting in pharmacoeconomic studies. Pharmacoeconomics.

[35] (2014). Centraal Bureau voor de Statistiek.

[36] Arkes H, Blumer C (1985). The psychology of sunk cost. Organ Behav Hum Dec.

[37] Hakkaart-van RL, Tan S, Bouwmans C (2011). Methoden en referentieprijzen voor economische evaluaties in de gezondheidszorg.

[38] Drost RM, Paulus AT, Ruwaard D, Evers SM (2014). Maastricht University.

[39] Bouwmans CA, Schawo SJ, Jansen DE, Vermeulen KM, Reijneveld SA, Hakkaart-van Roijen L (2012). iMTA Questionnaire Intensive Youth Care.

[40] Cloïn M, Kamphuis C, Schols M, Tiessen-Raaphorst A, Verbeek D (2011). The Hague: Sociaal Cultureel Planbureau.

[41] Jellinek.

[42] Killip S, Mahfoud Z, Pearce K (2004). What is an intracluster correlation coefficient? Crucial concepts for primary care researchers. Ann Fam Med.

[43] Drummond M, O'Brien B, Stoddart G, Torrance G (2005). Methods for the Economic Evaluation of Health Care Programmes.

[44] Cramer D, Howitt D (2004). The Sage Dictionary of Statistics: A Practical Resource for Students in the Social Sciences.

[45] (2015). Centraal Bureau voor de Statistiek.

[46] Byford S, Raftery J (1998). Perspectives in economic evaluation. BMJ.

[47] Jönsson B (2009). Ten arguments for a societal perspective in the economic evaluation of medical innovations. Eur J Health Econ.

[48] Knies S, Severens JL, Ament AJ, Evers SM (2010). The transferability of valuing lost productivity across jurisdictions. differences between national pharmacoeconomic guidelines. Value Health.

[49] Ahmad S (2005). Closing the youth access gap: the projected health benefits and cost savings of a national policy to raise the legal smoking age to 21 in the United States. Health Policy.

[50] Blankers M, Koeter MW, Schippers GM (2010). Missing data approaches in eHealth research: simulation study and a tutorial for nonmathematically inclined researchers. J Med Internet Res.

[51] Eysenbach G (2005). The law of attrition. J Med Internet Res.

[52] McKay HG, Danaher BG, Seeley JR, Lichtenstein E, Gau JM (2008). Comparing two web-based smoking cessation programs: randomized controlled trial. J Med Internet Res.

[53] Shahab L, McEwen A (2009). Online support for smoking cessation: a systematic review of the literature. Addiction.

[54] Webb TL (2009). Commentary on Shahab & McEwen (2009): Understanding and preventing attrition in online smoking cessation interventions: a self-regulatory perspective. Addiction.

[55] West R, Hajek P, Stead L, Stapleton J (2005). Outcome criteria in smoking cessation trials: proposal for a common standard. Addiction.

[56] Acock A (2005). Working with missing values. J Marriage Fam.

[57] Sobell LC, Cellucci T, Nirenberg TD, Sobell MB (1982). Do quantity-frequency data underestimate drinking-related health risks?. Am J Public Health.

[58] Devlin N, Parkin D (2004). Does NICE have a cost-effectiveness threshold and what other factors influence its decisions? A binary choice analysis. Health Econ.

[59] Neumann PJ, Cohen JT, Weinstein MC (2014). Updating cost-effectiveness--the curious resilience of the $50,000-per-QALY threshold. N Engl J Med.

[60] Evers SM, Hiligsmann M, Adarkwah CC (2015). Risk of bias in trial-based economic evaluations: identification of sources and bias-reducing strategies. Psychol Health.

[61] de Vries H, Logister M, Krekels G, Klaasse F, Servranckx V, van Osch L (2012). Internet based computer tailored feedback on sunscreen use. J Med Internet Res.

[62] Kohl LF, Crutzen R, de Vries NK (2013). Online prevention aimed at lifestyle behaviors: a systematic review of reviews. J Med Internet Res.

