# Supplementary material for: A Web-Based Computer-Tailored Alcohol Prevention Program for Adolescents: Cost-Effectiveness and Intersectoral Costs and Benefits
Source: J Med Internet Res. 2016 Apr 21;18(4):e93. doi: 10.2196/jmir.5223 (PMC4858595; doi:10.2196/jmir.5223)
Supplement: Multimedia Appendix 1 [file jmir_v18i4e93_app1.pdf]

**Cost category**	**Unit**	**Maximum amount**
Health care sector		
General practitioner	Contacts	60
Emergency care	Contacts	40
Hospital stays	Days	120
Ambulance rides	Rides	20
Mental health care	Contacts	60
Educational sector		
School absenteeism	Days	120
Attendance officer	Contacts	40
Labor & social security		
Work absenteeism	Days	120
Household & leisure		
Failure to perform household activities	Days	120
Failure to perform other activities	Days	120
Youth and family center	Contacts	40
Family care	Contacts	40
Criminal justice system		
Police services	Contacts	40
Youth police services	Contacts	40
Court proceedings	Amount	40
Child protection services	Contacts	40
Child health protection services	Contacts	40
Substance use		
Cigarettes	Packs	360
Soft drugs	Joints etc.	360
Hard drugs	Pills etc.	360
