# Supplementary material for: A Web-Based Computer-Tailored Alcohol Prevention Program for Adolescents: Cost-Effectiveness and Intersectoral Costs and Benefits
Source: J Med Internet Res. 2016 Apr 21;18(4):e93. doi: 10.2196/jmir.5223 (PMC4858595; doi:10.2196/jmir.5223)
Supplement: Multimedia Appendix 2 [file jmir_v18i4e93_app2.pdf]

Male subgroup

Female subgroup

Younger adolescents subgroup

Older adolescents subgroup

Lower educational level subgroup

Higher educational level subgroup

Religious subgroup

Non-religious subgroup

Dutch ethnicity subgroup

Other ethnicity subgroup
